# Histone Lysine Methylation Modification and Its Role in Vascular Calcification

**DOI:** 10.3389/fendo.2022.863708

**Published:** 2022-06-16

**Authors:** Ye-Chi Cao, Su-Kang Shan, Bei Guo, Chang-Chun Li, Fu-Xing-Zi Li, Ming-Hui Zheng, Qiu-Shuang Xu, Yi Wang, Li-Min Lei, Ke-Xin Tang, Wen-Lu Ou-Yang, Jia-Yue Duan, Yun-Yun Wu, Muhammad Hasnain Ehsan Ullah, Zhi-Ang Zhou, Feng Xu, Xiao Lin, Feng Wu, Xiao-Bo Liao, Ling-Qing Yuan

**Affiliations:** ^1^ National Clinical Research Center for Metabolic Diseases, Department of Metabolism and Endocrinology, The Second Xiangya Hospital, Central South University, Changsha, China; ^2^ Department of Cardiovascular Surgery, The Second Xiangya Hospital, Central South University, Changsha, China; ^3^ Department of Radiology, The Second Xiangya Hospital, Central South University, Changsha, China; ^4^ Department of Pathology, The Second Xiangya Hospital, Central South University, Changsha, China

**Keywords:** epigenetics modification, histone lysine methylation, histone lysine methyltransferases (HKMTs), vascular calcification, signalling pathways

## Abstract

Histone methylation is an epigenetic change mediated by histone methyltransferase, and has been connected to the beginning and progression of several diseases. The most common ailments that affect the elderly are cardiovascular and cerebrovascular disorders. They are the leading causes of death, and their incidence is linked to vascular calcification (VC). The key mechanism of VC is the transformation of vascular smooth muscle cells (VSMCs) into osteoblast-like phenotypes, which is a highly adjustable process involving a variety of complex pathophysiological processes, such as metabolic abnormalities, apoptosis, oxidative stress and signalling pathways. Many researchers have investigated the mechanism of VC and related targets for the prevention and treatment of cardiovascular and cerebrovascular diseases. Their findings revealed that histone lysine methylation modification may play a key role in the various stages of VC. As a result, a thorough examination of the role and mechanism of lysine methylation modification in physiological and pathological states is critical, not only for identifying specific molecular markers of VC and new therapeutic targets, but also for directing the development of new related drugs. Finally, we provide this review to discover the association between histone methylation modification and VC, as well as diverse approaches with which to investigate the pathophysiology of VC and prospective treatment possibilities.

## 1 Introduction

Vascular calcification (VC) is a pathological condition prevalent in persons with atherosclerosis, hypertension, chronic kidney disease and diabetic vascular disease. It presents a substantial risk factor of cardiovascular disease (CVD) occurrence and death, as well as an independent predictor of CVD occurrence (1). According to the location of calcified plaque formation and development, VC is classified as either intimal calcification or medial calcification (2). Intimal calcification is evident in atherosclerotic plaques (3), where vascular cells undergo metamorphosis and functional changes that encourage the creation of calcium and phosphorus crystals in the lipid necrosis nuclei of atheromatous plaques. Medial calcification is seen in distal arteries (4), which can cause vascular compliance to decrease and pulse pressure to rise, resulting in cardiac insufficiency. Both forms of calcification can occur individually or simultaneously in patients with CKD. VC is primarily caused by phenotypic changes of vascular smooth muscle cells (VSMCs), a highly changeable process analogous to bone development. VC is caused by a variety of factors, including oxidative stress, inflammatory response, autophagy (5), vesicle production, vascular injury, high calcium and phosphorus levels, and a lack of calcification inhibitory factors, all of which contribute to mineral deposition in the extracellular matrix and to VC (6, 7). Various studies have been carried out to understand the intricate molecular pathways that govern gene expression and protein function, in order to slow down the process of VC and identify appropriate treatments for cardiovascular disease.

Post-translational regulations are covalent modifications that occur after protein synthesis and are important targets for the regulation of signalling pathways. Histone lysine methylation modifications are one of these, mediated by histone lysine methyltransferases (HKMTs), which may operate on both histones and non-histones and play important roles in many biological processes through heterochromatin formation, transcriptional control, and so forth. A range of pathological conditions can be caused by abnormalities in lysine methylation modification, and studies have revealed that lysine methylation modification is intimately linked to VC formation and development.

This review summarizes well-known mechanisms of VC, followed by recent updates regarding lysine methylation–associated VC pathogenesis and related pathways. Further studies are needed to uncover the complicated interactions during lysine methylation modification and to achieve a breakthrough in therapy for VC-associated diseases. These complex and cross-talking mechanisms closely support the postulate that VC is affected by histone lysine methylation. Understanding specific VC pathologies associated with HKMTs and exploring the potential application of epigenetics to treatment would be of profound significance.

## 2 Mechanisms of VC

VSMCs exist in and can interconvert between two phenotypic states, namely the contractile (differentiation) and synthetic (de-differentiation) states (8). The change from a contractile to a synthetic state, which is a pivotal phase at the onset of severe vascular proliferative diseases, results in increased VSMC proliferation and migration, extracellular matrix secretion and synthesis, and the formation of neointimal membranes. Bone-related genes, such as bone morphogenetic proteins (BMPs), runt-related transcription factor 2 (Runx2) (8), and osteocalcin can have increased expression followed by transition. Abnormal calcium and phosphorus metabolism, inflammation and oxidative stress, pro- and anti-calcification factor imbalance, and autophagy, all of which interact to influence the formation and progression of VC, have been identified as the etiology of VC. In short, multiple factors that contribute to alterations of body homeostasis have been shown to be strongly associated with the onset and progression of VC.

### 2.1 Abnormal Metabolism of Calcium and Phosphorus

Abnormal mineral homeostasis caused by elevated calcium and phosphorus concentrations can mediate VC. High phosphate in serum leads to VSMC calcification *in vitro* (9) and to coronary artery calcification in human (10), suggesting that phosphate plays a critical role in the pathophysiology of VC, and the degree of calcification is dose-dependent on phosphorus concentration. Meanwhile, it is worth noting that under conditions of normal phosphorus concentration, the degree of VC in VSMCs can be upregulated when the calcium concentration increases (11), and high levels of calcium can lead to the formation and development of hydroxyapatite crystals in the VSMCs. High phosphate upregulates Pit1 to raise intracellular levels of inorganic phosphate, followed by downregulation of calcification inhibitors, release of extracellular vesicles, remodelling of the extracellular matrix and apoptosis of VSMCs (9). Eventually, various signalling pathways trigger phosphate-induced osteo-/chondrogenic transdifferentiation of VSMCs, leading to VC, and the main feature of the osteogenic signalling pathway in VC is the upregulation of Runt-related transcription factor 2 (Runx 2). In addition, previous studies showed that high extracellular phosphate levels induced apoptosis and necrosis of VSMCs, and so apoptotic bodies released from VSMCs could serve as a nidus for calcium phosphate deposition (12). Briefly, the disturbance in calcium and phosphorus metabolism has a relatively direct role in the progression of VC, and attention to the physiopathological processes associated with calcium and phosphorus metabolism may provide direction for the treatment and prevention of VC.

### 2.2 Inflammation and Oxidative Stress

The inflammatory response pathway is an important venue associated with pathogenesis of VSMCs calcification (13), and the progression of VC is aided by many inflammatory cells and factors. Interleukin-1 beta (IL-1β) stimulates VSMC calcification *in vitro* (14). Interleukin-6 (IL-6)/soluble interleukin-6 receptor (sIL-6R) complexes induces VSMC transformation into an osteoblast phenotype (15). IL-11 plays an important role in VSMC phenotype switching and vascular inflammation (16). When macrophages are exposed to calcium or phosphate nanocrystals, they release inducible nitric oxide synthase (iNOS) and tumour necrosis factor (TNF), implying that inflammatory immune cells are recruited to the calcification site (17). According to previous work, macrophages from various subsets can undergo polarity drift, meaning that macrophages from different subsets can turn into one another. For example, under specific conditions, M1 and M2 may bio-transform into each other. Macrophages can promote VC through diverse mechanisms, such as the release of reactive oxygen species, pro-inflammatory cytokines and matrix vesicles (MVs) (18). This process is regulated by cartilage oligomeric matrix protein (COMP), which can polarize macrophages into M1 phenotypes (i.e., they tend to be osteogenic phenotypes), and inhibits macrophages’ differentiation into M2 and osteoclast-like cells (19). M1 can directly release oncostatin M (OSM) to promote the differentiation of VSMCs into osteoblastic phenotypes through the JAK3-STAT3 pathway (20), whereas M2 can secrete anti-inflammatory factors, as well as phagocytize necrotic fragments and apoptotic cells, to prevent the formation of calcified nucleation sites (21). However, the persistent state of chronic inflammation caused by M1 may also impair the normal development and transformation of VSMCs into osteoblasts. The macrophages can produce BMP2 as well as Runx2, and this ability can be maintained in aortic plaques (18). This process reveals a novel therapy avenue in which controlling the M1-to-M2 transition and reducing inflammation may help to reduce VC. Researchers found that inhibition of Runx2 regulation mediates the anti-calcification effect by inhibiting the inflammation-associated NF-κB pathway (22), suggesting a strong link between inflammation and calcification in the pathogenesis of VC. Thus, these discoveries indicate a strong causal relationship between inflammation and VC, and the research of VC should be accompanied by attention to inflammatory indicators, suggesting that certain inflammatory indicators can be used as tools to evaluate VC.

### 2.3 VC Activator

#### 2.3.1 FGF23

FGF23 is produced by osteocytes, which have been found to be one of the most powerful phosphomodulators (23) and an important inducer of VC. It acts in collaboration with the transmembrane protein Klotho as a cofactor, primarily regulating the metabolic balance of blood phosphorus and vitamin D in the body (24). Klotho has been identified as an anti-aging gene (25) and Klotho protein has anti-aging and cardiovascular protective effects. Lim et al. discovered that human VSMCs express Klotho protein (26). They also discovered that Klotho expression was significantly reduced and Runx2 was increased in vascular tissues, and that Klotho protein inhibited the transdifferentiation of VSMCs into osteoblasts by inhibiting phosphorus uptake by cells. Klotho deficiency can promote the osteogenic phenotype of VSMCs by regulating phosphorus uptake in VSMCs, through the induction of the sodium-phosphorus cotransporter Pit1/2 (27). Numerous experiments have shown that Klotho deficiency is an important causal factor in VC. Elevated levels of Klotho may regulate phosphorus and calcium homeostasis *in vivo*, either directly in the kidney and vascular cells or indirectly (28). The effect of Klotho on calcification may be associated with the classic Wnt/-catenin pathway (29, 30), and a study involving stem cells demonstrated Klotho may act as a Wnt antagonist and immunoprecipitates with a number of Wnt isoforms, including Wnt1, Wnt3, Wnt4 and Wnt5a (31). Hum et al. found that upregulation of Klotho alleviated VC (32); for example, intermedinl-53, a member of the adrenomedullin family, has been shown to reduce the degree of VC by activating the cyclic adenosine monophosphate/protein kinase A (cAMP/PKA) signalling pathway (33). However, Lindberg (34) could not detect mRNA expression of FGF23 or its coreceptor, Klotho, in human or mouse VSMCs, nor normal or calcified mouse aorta. There still exists controversy as to whether VSMCs express Klotho, and whether Klotho is involved in the development and progression of VC. Therefore, insights into FGF23 may provide a new indication for the development of VC, which may provide a direction for treatment and prevention, while a complete understanding of this pathway and other roles in VC remain to be confirmed.

#### 2.3.2 BMP

BMPs are powerful osteogenic differentiation activators, and were discovered in calcified VSMCs. BMP-2 and BMP-4, in particular, are intimately linked to VC among BMP family members. BMP-2 may promote VC by activating muscle segment homeobox2 (MSX2) and inhibiting matrix Gla protein (MGP), and may also promote apoptosis of VSMCs (35). BMP-2 and MSX2 can activate the Wnt/β-catenin pathway, one of the major osteo-inductive signalling pathways in VC, and then induce VSMC calcification (36). Nuclear factor-kappa B ligand (RANKL) promotes VC by inducing the release of BMP-2 from human aortic endothelial cells, which, in turn, acts in a paracrine manner on the adjacent human aortic smooth muscle cells to increase osteoblastic activity (37). In vascular media, BMP2 was observed to act through the type III sodium-dependent phosphate cotransporter, Pit1, and downregulate microRNA-30b and 30c (38), resulting in an increased expression of Runx2, calcium deposition, and mineralization to accelerate medial or intimal calcification. BMP‐2 signalling is involved in the maintenance of the contractile phenotype and has been shown to inhibit VSMCs proliferation and neointimal hyperplasia (39). Serum BMP4 levels are higher in patients with chronic renal disease and coronary artery disease, and they are independently and positively linked with coronary artery calcification indices (40). BMP-4 may participate in leptin-induced calcification of VSMCs *via* ERK1/2/RANKL/BMP-4 and PI3K/Akt/RANKL/BMP-4 signalling pathways (41, 42). In addition, BMP4 can promote foam cell production, inhibit lipid carrier expression and lipid export, and contribute to atherosclerosis through the BMPR1/2/Smad1/5/8 signalling pathway (43). These discoveries systematically and clearly illustrate the role of BMP as an activator in VC, which affects the onset and progression of the disease.

In addition to the above two activators of calcification that are more associated with histone lysine methylation modifications, there exist many other risk factors that promote calcification, including osteocalcin (OC) (44), alkaline phosphatase (ALP) (45), osteopontin (OPN) (46), PTH (47) and Cathepsin K (48). Together, these activators of calcification are involved in key aspects of the VC signalling pathway and focusing on these activators to find HKMTs which produce inhibitory effects is an important therapeutic direction for the alleviation and treatment of VC.

### 2.4 VC Inhibitors

As the external environment changes, the corresponding decrease in calcification inhibitors and functional deficiencies can exacerbate VC. There are natural inhibitors of calcification including osteoprotegerin (OPG) (49), matrix Gla protein (MGP) (50), fetuin-A (Fet-A) (51), BMP7 (35) and pyrophosphate (PPi) (52). Paloian identified a novel protein involved in the bone-vascular axis, osteosclerin (OPN), which is mainly secreted by osteoblasts and chondrocytes. As a specific and regulated negative regulator of the Wnt pathway, osteosclerin plays a protective role in the development of VC (53). These findings about VC inhibitors provide some directions for the identification of new therapeutic targets, suggesting analysis of the differential expression of these inhibitors may provide novel outlooks on diagnosis and therapy.

### 2.5 Disturbed Autophagy Regulation

Autophagy is a cell protective mechanism occurring through removal of mistake proteins, damaged organelles or unwanted metabolites (54), which is vital to maintain normal VSMC function (55–57). However, excessively activated autophagy can induce autophagic death of VSMCs (58). For example, hyperphosphatemia can induce calcification by promoting osteogenic transformation of VSMCs through activating abnormal autophagy (54, 59). Cell death by apoptosis or necrosis leads to the release of apoptotic bodies, or necrotic debris, which may act as nucleation sites for calcium phosphate deposition and further aggravate calcification. In contrast, research showed that autophagy could counteract against ROS-induced VC at high Pi concentrations *in vitro* (59). In addition, emerging evidences indicates that autophagy also regulates extracellular matrix homeostasis and mitigates VC process (60). To summarise, whether autophagy is a protective or harmful mechanism in VC pathology remains controversial. The idea of modulating autophagy offers an attractive direction to treat or prevent VC. For example, oestrogen-induced autophagy inhibits the osteogenic differentiation of VSMCs and arterial calcification *via* the ERα pathway (57). Therefore, further studies are required to investigate autophagy and their significance in pathophysiology of VC.

## 3 Histone Lysine Methylation

**Figure 1:**
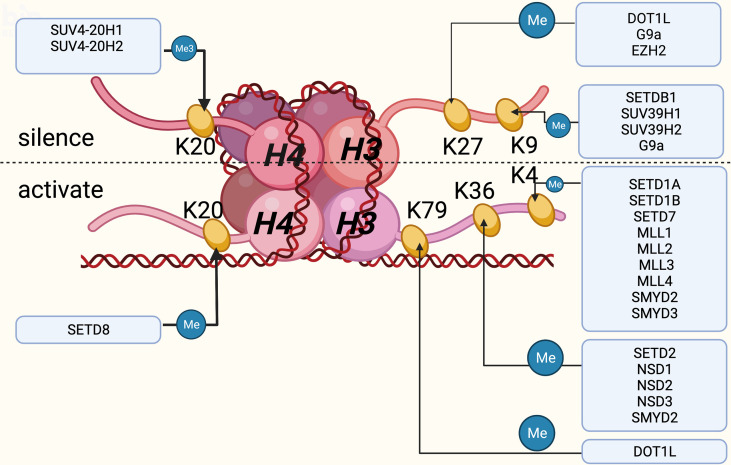
Histones and the DNA wound around the histones together constitute the nucleosomes, which are the main structural elements of chromatin, mainly composed of H2A, H2B, H3 and H4. Histone lysine methylation occurs mainly at H3 and H4, of which six sites are currently well studied. This figure summarizes the role of lysine methylation activation or inhibition at these six sites and the frequently regulated enzymes for them. Created with BioRender.com.

In eukaryotes the nucleosome is the main structural element of chromatin, which consists of DNA and a core histone octamer including two copies of H2A, H2B, H3 and H4, with histone H1 acting as a junction between the nucleosome and DNA (61). Histone acetyltransferase, histone methyltransferase and histone phosphotransferase can all produce post-translational modifications, of which histone lysine methylation is one of the most characterized post-translational modification. With more clues uncovered, lysine methylation modification plays a significant role in the progress of VC. Mechanistically speaking, only lysine and arginine were thought to be the locations of histone methyltransferase action, based on early investigations on a vast number of protein sequences, and the N-terminal tails of histone residues can be methylated once or repeatedly, resulting in monomethylation, dimethylation or trimethylation. HKMTs are a family of proteins that include the SET domain, named after the first three genes that expressed it: Su(var)3-9, enhancer of zeste [E(z)] and trithorax (trx) (62). Apart from Dot1 enzyme (63), which methylates H3K79, all HKMTs have a SET domain. Histone lysine methylation is directly linked to chromatin concentration and gene silencing (64), and can play a role in physiological and pathological states *via* a variety of pathways. Thus, HKMTs can be summarized into two categories based on their different functions on the substrate, one activating and the other inhibiting. H3K4, K26, K36, K79 and H4K12 methylations are mostly engaged in gene activation, whereas H3K9, K27, K56, H4K5, and K20 methylations are involved in gene silencing (**Figure 1**).

## 4 HKMTs Substrates Lysine From Histone to Control VC

### 4.1 Transcriptional Activation-Associated Histone Lysine Methylation Modifications in VC

#### 4.1.1 H3K4 Locus Methylation and its Potential Role in Facilitating VC

H3K4 methylation is recognized as a marker of gene transcriptional activation. H3K4 methyltransferases, also known as MLL (mixed lineage leukaemia) family proteins, include MLLl, MLL2, MLL3, MLLL4, SET1A, SET1B, SETD7, SMYD2 and SMYD3. SMYD2 methylates histone H3K4 and H3K36 with the SET-dependent manner. TGF -β induces increased deposition of the extracellular matrix when the level of H3K4me methylated by SMYD2 increases (65), and TGF-β1 or BMP2-stimulated valvular endothelial cells transform into osteoblast-like cells by increasing ALP expression, eventually resulting in VC (66). SMYD3 catalyzes dimethylation and trimethylation of H3K4 to form H3K4me2 and H3K4me3 near the promoter regions of target genes, which usually serve as transcriptional activators involved in promoting cell growth (67). SMYD3 promotes vascular cellular senescence by binding to the promoter region of p21 gene, implicated in cell cycle arrest and cellular senescence *via* H3K4 methylation (68). Other research also shows that SMYD3 can bound to promotors of PARP16 through increased H3K4me3 levels, to mediate vascular senescence and ER stress existing in cell models (69). Research on SMYD3 in VC has not yet been carried out, but there are many common points of contact between vascular senescence and calcification in terms of their mechanisms, which can provide a novel direction for the study of VC. Increasing evidence suggests that SETD7 plays a critical role in a number of physiological and pathological processes, such as metabolism, immunity, vascular pathology and cancer (70). Hypoxia-inducible factor 1α (HIF-1α) is a transcription factor upregulated by hypoxia, and SETD7 can inhibit its transcriptional activity by methylating H3K4 (71). HIF-1α can upregulate Runx2 to induce VC (72) and plays a critical role in Pi-induced VC (73). However, our understanding of the relationship between H3K4 methylation and VC is rudimentary and is based on the association of other vascular pathological alterations and VC in previous studies. In conclusion, H3K4 methylation contributes to an enhanced interpretation of VSMCs physiology and pathology.

Current studies on MLL family methylation modifications are mainly in the tumour area, with few reports investigating the VC area. It is noteworthy that MLL1 facilitates the proliferation of myoblasts by epigenetically regulating Myf5 *via* mediating H3K4me3 on its promoter (74). Also, SETD7 has been shown to be associated with transcriptional activation of myogenic differentiation genes, such as MYOD, MYOGENIN, MHC and MCK, *via* H3K4 methylation (75), providing an insight into the mechanisms of HKMTs in VSMC pathophysiology. These discoveries further support the potential role of HKMTs acting on H3K4 in VC progression.

#### 4.1.2 H3K36 Locus Methylation and its Potential Associated Role in VC

H3K36 plays an important role in transcriptional elongation, and its methylation modifications may be seen in abundance in the coding areas of transcriptionally active genes (76). H3K36me1 is considered an intermediate modification without any significant role, whereas H3K36me2 and H3K36me3 each play a major role in histone modifications (77). Zhou et al. revealed that NSD2 could serve a new function in the etiology of pulmonary arterial hypertension by elevating the H3K36me2 level, which regulates trehalose metabolism and autophagy (78). According to a genome-wide investigation, DNMT3A binding and activity co-localize with H3K36me2 at non-coding areas of euchromatin, and NSD1-mediated H3K36me2 is required for the recruitment of DNMT3A and the maintenance of DNA methylation at intergenic regions, showing the intrinsic interactions of histone and DNA in epigenetic modifications (79). SMYD2 inhibits macrophage activation by facilitating H3K36 dimethylation at TNF and IL6 promoters, and increased SMYD2 expression decreases the production of pro-inflammatory cytokines including IL-6 and TNF. Then, as a result of enhanced TGF-production and reduced IL-6 release, macrophages with elevated SMYD2 expression promote regulatory T cell differentiation (80). Although no direct links between VC and H3K36 methylation modifications have been discovered, several studies in other diseases have shown that H3K36 methylation modifications are involved in the regulation of VC risk factors, suggesting that there may exist potential mechanisms for researchers to investigate.

#### 4.1.3 H3K79 Locus Methylation and its Potential Associated Role in VC

H3K79me is a marker of activated chromosomes and is significantly expressed in areas with high gene transcriptional activity (81). Disruptor of telomeric silencing 1-like (DOT1L) is a methyltransferase that acts on lysine 79 of histone H3 (H3K79). In addition to regulating transcriptional activation of certain genes, DOT1L is also involved in DNA repair, cell differentiation, and cell cycle regulation (82, 83). In the G1 phase, DOT1L-deficient cells experience irreversible cell cycle arrest, resulting in premature senescence (74). Increased levels of SIRT1 caused by AMPK activation can lead to an increase in H3K79me3 *via* DOT1L upregulation, and then to H3K79me3-induced up-regulation of SIRT3 levels, enhancing mitochondrial biogenesis function as well as delaying vascular senescence (84). Vascular senescence and VC have many common contributors to the mechanism, and this has given researchers new ideas as to whether H3K79 methylation plays a similar role in VC. In a nutshell, these results present the probably function of H3K79 associated enzymes in VC.

### 4.2 Transcriptional Repression-Associated Histone Lysine Methylation Modifications in VC

#### 4.2.1 H3K9 Locus Methylation–Associated Protective Mechanisms of VC

H3K9 methylation plays an important role in X-chromosome silencing, heterochromatin formation, DNA methylation and transcriptional regulation. Researchers have confirmed that methylation of H3K9 was significantly reduced in atherosclerotic plaques in SMCs and inflammatory cells (85), which illustrates the important role of H3K9 methylation in the activation of SMCs in atherosclerosis and macrophages. G9a, SUV39H2, SETDB1 and SUV39H1 are the primary methyltransferases at this locus. G9a is the main euchromatin H3K9 methyltransferase, catalyzing monomethylation and dimethylation of H3K9 (86) in euchromatic regions, and is involved in trimethylation of H3K9 (87). The methylation of H3K9 by G9a is mostly associated with gene silencing. G9a can epigenetically silence Klotho expression by monomethylating H3K9 on the Klotho promoter (88), whereas silencing G9a has the reverse effect, apparently reversing the repressive effect of Klotho expression (89). As a result, inhibiting G9a expression can control the development of VC *via* FGF23/Klotho axis, which may provide an important therapeutic strategy for VC and deserves consideration. Vascular inflammation is an important contributor to the development of VC, and SUV39H1 exerts anti-inflammatory effects in the vascular inflammatory response by inhibiting the transcription of downstream target genes, such as NOS (90), and modulating the NF-κB signalling pathway (91). Whether or not this role in inhibiting the development of inflammation delays VC deserves further investigation. Therefore, future studies are required to explore these mechanisms in detail. As more studies are conducted, targeted therapies aimed at H3K9 methylation may provide clinicians with a new therapy to attenuate cardiovascular diseases.

#### 4.2.2 H3K27 Locus Methylation–Associated Protective Mechanisms of VC

H3K27 is associated with the transcriptional repression of genes and can result in the inactivation of X-chromosomes (92). The results of several studies suggest that the overall level of H3K27me3 modification is reduced in VSMCs containing atherosclerotic plaques (85, 93). EZH2 is regarded as a transcriptional suppressor that targets genes to alter cell biological behaviour, by generally silencing the expression of the target gene (94). EZH2 acts on H3K27 and catalyzes methylation to form H3K27me3, which affects chromatin configuration and genome stability. In addition to generating H3K27me3, EZH2 can also recruit DNA methyltransferase 1(DNMT1) (95) in the promoter region of target genes to directly silence their expression, and DNA damage can, to a certain extent, promote VC (96). Several studies have confirmed that EZH2 expression is abnormally elevated in cancer tissues and is positively correlated with the degree of malignancy of cancer (97). Apart from functioning in cancer, EZH2 was also involved in several signalling pathway modulations, including playing a negative regulatory role in the process of muscle cell differentiation and the pathophysiologic processes of VC (98). EZH2 plays a critical role in the differentiation of skeletal muscle cells in myoblast and myosatellite cells through miR-101a, which promotes skeletal muscle cell differentiation directly through EZH2 (99), instead of the terminal stage of differentiation (100). Reduced EZH2 expression increases expression of ATG5 and ATG7 and activates the MEK–ERK1/2 signalling pathway, which induces excessive autophagosome formation and then leads to VSMC loss (101). EZH2 promotes triple methylation of H3K27 in the promoter region of ATP-binding cassette transporter A1 gene and represses its transcriptional expression, which is associated with atherosclerosis pathology and ultimately leads to the appearance of VC. Han et al. discovered that H3K27me3 catalyzed by EZH2 was abundant in the promoter region of the Klotho gene, due to directly binding to the Klotho promoter, and suppressed Klotho gene expression (102) which also leads to VC. H3K37 methylation changes can act on macrophages to promote the process of VC, in addition to influencing the phenotypic alteration of VSMCs. Increased H3K27me3 levels on the promoter of nuclear factor of activated T cells type c-1 (NFATc-1) causes epigenetic impairment of the NFATc-1 gene, resulting in macrophages in the vicinity of calcium deposits being phenotypically deficient and unable to resorb calcification (103). The methylation modification H3K27 has been shown to be a risk promoter for VC, and considering H3K27 as a target for inhibition of methylation modification or demethylation of H3K27me3 will provide new ideas for the alleviation and treatment of VC.

#### 4.2.3 H4K20 Locus Methylation–Associated Protective Mechanisms of VC

H4K20 methylation plays key roles in DNA replication, gene damage repair, and silenced heterochromatin (104), which was once thought to be a methyltransferase target for gene silencing. However, recent research suggests that H4K20-associated methylation may play a dual role in epigenetic regulation (105). H4K20me1 is linked to transcriptional activity and controls chromatin condensation, whereas H4K20me3 is a hallmark of suppressed heterochromatic areas and is linked to transcription repression and transposon activity (106). SETD8 is the only methyltransferase known to catalyze the monomethylation of H4K20, and can govern gene transcription, maintain genomic integrity and regulate cell-cycle progression by monomethylating H4K20. The vast majority of di- and tri-methylation modifications are mediated by the SUV4-20H1 and SUV4-20H2 enzymes (107). Besides acting on lysine, SET8 also can methylate non-histone proteins such as TWIST and p53 (105). On the one hand, SET8 binds directly to and inactivates the target gene by monomethylating H4K20, resulting in the inhibition of its downstream pathways in the pathogenesis of some diseases (108); on the other hand, SET8/H4K20me1 is enriched in the promoter and coding regions of transcriptionally active genes, for example, mediating the transcriptional activation of Wnt target gene (109), which can regulate the expression of the target protein RUNX2 and, in turn, may regulate the phenotypic transformation of VSMCs (110). Furthermore, downregulation of SET8-mediated H4K20me1 is involved in the increasing modulation of PTEN expression, which mediates endothelial inflammation to generate VC (111). Yang et al. discovered that SET8 also acts as a protective epigenetic modifier on the TWIST promoters *via* its H4K20 monomethylation activity. When factors that contribute to the inhibition of SET8 expression, such as high phosphorus, TWIST target genes can be monomethylated to inhibit AKT expression by negatively regulating their transcription, and then Caspase-3 expression can be promoted, which, in turn, promotes apoptosis and increases the development of calcification in VSMCs (112, 113).

Although SET8 has a complex mechanism of action in H4K20 and non-histone catalysis, in terms of overall effect SET8 is significantly downregulated in the calcification model (114). The methylation modification of H4K20 is highly complex due to the different effects at different levels of methylation; therefore, researchers may focus on the specific role of SET8 in different pathways and the effects of other H4K20 methyltransferases on VC.

## 5 HKMTs Substrates Lysine From Non-Histone to Control VC

According to current research, HKMTs have a broad function in identifying methylated lysines on non-histone proteins. The area of non-histone methylation is still in its early stages, and the majority of applications identified thus far are p53-related. Furthermore, HKMTs catalyze a variety of non-histone proteins involved in the VC pathological process.

### 5.1 HKMTs Methylate p53 Lysine and Participate in VC

The p53 oncogene, in addition to being one of the most important tumour suppressors in cells, has recently been discovered to play a vital role in VC (115, 116). The HKMTs SET9, SMYD2, G9a and SET8 can methylate four of the six lysine sites at the C-terminus of the p53 protein to modulate its function (117). Consequently, p53 activity can be enhanced or lowered after methylation modification, and the response of p53-mediated transcription activation suppression depends on target genes. SET9 mono- and dimethylates p53K372 to regulate the expression of p53 target genes, and positively affects p53 stability (118, 119). SMYD2 was thought to be a transcription coactivator as it can deposit methyl groups on histones H3K4 and H3K36, both of which are epigenetic signatures of active transcription (120). However, unlike its action on histones, SMYD2 generally performs an inhibitory role in regulating non-histone proteins. For example, SMYD2 directly monomethylates p53 at K370 to inhibit its transactivation (121). In addition, SMYD2 was reported to act as an endogenous antagonist of p53-dependent cardiomyocyte apoptosis (122), which may have a connection to VSMCs. G9a dimethylates p53 at lysine 373 to inhabit p53 activity (123), and G9a inhibitors that restore p53 activity may act as therapeutic agents for treating specific diseases (124). SET8 inhibits apoptosis and cell cycle arrest, by monomethylating the lysine 382 site of p53 to p53K382me1 and decreasing p53 transcription (125). By regulating the p53/Bcl-2/caspase signalling pathway, SET8 can downregulate the expression of anti-apoptotic protein Bcl-2 and upregulate the expression of pro-apoptotic proteins Bax and Caspase3, thus participating in the regulation of calcification and apoptosis in VSMCs when its expression is reduced by various factors (126). Meanwhile, lysine methylation impairs the function of the Numb phosphotyrosine-binding (PTB) domain, detaches Numb from p53, and prevents it from performing its pro-apoptotic role (127). The mTOR pathway may affect VSMC senescence through upregulation of p53/p21/p16 (128). Upregulation of p53/p21/p16 by the mTOR pathway impacts VSMC senescence (52), and downregulation of p53 expression by activation of the ROS/p53/p21 pathway has also been discovered to slow down the process of vascular aging (129). Using p53 knockout or p21 knockout mice, however, it was discovered that the atherosclerotic lesion developed faster than the wild type, even though DNA damage and VSMC death were reduced compared to the control. This could be due to the multiple functions of the p53 and p21 genes, implying that there may be another unknown mechanism in the p53-associated VC. Researchers have identified HKMTs as p53-modifying enzymes and have suggested analyzing how methylation may help maintain normal physiological function to regulate different p53 functions, in search of a dynamic equilibrium that can both inhibit apoptotic pathways and reduce VC. Further, the influence of p53 is likely an important area of future research, as their methylation state could affect different substrates to modulate the dysregulation of multiple pathological states and restore them to a relatively normal state.

### 5.2 Heat Shock Proteins Are Associated With VC and Can Be Methylated by HKMTs

Several post-translational modifications are also present in HSPs, including methylation modifications. The current research found that the dimethylation of HSP70 lysine at position 561 was catalyzed by SETD1A (112), while HSP90AB1 lysine at position 531 and lysine at position 574 were dimethylated by SMYD2 (130). Similarly, SMYD2 methylates Hsp90 in muscle to maintain titin stability and muscular function (131). Yao et al. illustrated that HSP70 mediated the procalcific effect on calcifying vascular cells by binding to MGP and enhancing BMP activity, showing a potential connection between cellular stress, inflammation, and BMP signalling (132). Together, these studies demonstrated that HKMTs can methylate HSPs and HSPs serve a role in the VC progress-related pathways, so what requires further confirmation is the specific mechanism of HSPs in VC and whether HKMTs play an HSP-associated role in VC.

### 5.3 p65 Is Involved in VC and Plays a Dual Role *via* Different HKMTs

P65 is one of the most important components of the transcription factor nuclear factor-κB (NF-κB), which regulates the expression of a wide variety of genes (133). NF-κB -p65 activated by phosphorylation, in response to various factors, promotes transcription of target genes (134). NSD1 was discovered to catalyze the methylation of p65 K218/K221me, enhancing the transcriptional activity of the NF-κB pathway (135). Increased circulating levels of other inflammatory factors, such as TNF, IL-6 and NF-κB, can promote calcification in VSMCs (136), and interleukin 1β (IL-1β) induces osteogenic transformation of VSMCs through the activation of the NF-κB/p53/p21 pathway, ultimately leading to VC (137). Six methylated K sites, K37, 218, 221, 310, 314, and 315, have been identified on the p65 subunit of NF-κB (138). Yang and colleagues reported that p65 is monomethylated by SET9 on K314 and 315, resulting in inhibition of NF-κB action by inducing the proteasome-mediated degradation of the p65 promoter (139). However, methylation of SET9 at the p65K37 site exhibited the opposite effect, and could activate the NF-κB pathway (140). SETD6 monomethylates p65 on K310, leading to the induction of a repressed state of NF-κB target genes through the binding of G9a-like protein (141). Taken together, the NF-κB pathway is critical in VC and in the development of many diseases. Various HKMTs act on p65 in this pathway to inhibit or promote pathogenesis, and it is worthwhile to investigate how to reduce p65-related methylation activation, and, thus, control the NF-κB pathway to reduce the occurrence of VC.

### 5.4 HKMTs Catalyze ERα Lysine and Control Its Downstream Function

Studies have shown that different HKMTs operate at different locations on the oestrogen receptor alpha (ERα) and thus show different effects in physiopathological processes. SETD7 catalyzes the monomethylation of ERα lysine position 302 (ERαK302me1) to stabilize the ERα protein, which is necessary for the efficient recruitment of ER to its target genes and the activation of an oestrogen-driven transcriptional response (142). SMYD2 catalyzes ERK266me1 and prevents ER binding to chromatin, inhibiting ER target gene activation (143). G9a methylates ERα at K235, attracting the PHF20/MOF complex to deposit histone acetylation and boosting gene activation (144). Oestrogen inhibits VC by modulating the receptor activator of nuclear factor-kappa B (RANK) and RANKL signalling pathways (145). Runx2, a major osteogenic transcription factor expressed in calcified atherosclerotic plaques, can be inhibited by estradiol in osteoblasts (132). Meanwhile, McRobb et al. illustrated an opposite effect, that oestrogen can promote calcification in advanced atherosclerotic lesions by promoting the differentiation of VSMCs to osteoblast-like cells, and this process could be augmented by inhibition of ERα or ERβ activity (146). These findings provide some directions for the identification of novel ERα methylation targets associated with VC in the later stage, and for the development of novel therapies. Researchers may further investigate how ER methylation can play a role in reducing VC, based on numerous studies that have demonstrated the preventive effects of oestrogen in cardiovascular disease.

### 5.5 Methylated MAPK Participates in Various Diseases

The mitogen-activated protein kinase (MAPK) route, the P13K system, and the cyclic adenosine phosphate (CAMP) pathway are all implicated in controlling the phenotypic transition of VSMCs. RAS/RAF/MEK/ERK1/2 is a typical MAPK signal transduction pathway (147) and MAPK/ERK has a verified relationship with vascular dysfunction (148). SMYD3 methylates MAP3K2 at lysine 260, increasing MAPK signalling (149), and SMYD3 overexpression is associated with poor prognosis in a variety of diseases (150). Overall, we believe that the impact of MAPK lysine methylation should not be overlooked in the VC research. Exploration of MAPK methylation modifications could explain the related pathway in VC and become a new target with which to overcome VC in the future.

## 6 HKMTs Catalyze Lysine Substrates to Participate in the Mechanism of Vascular Calcification

**Figure 2:**
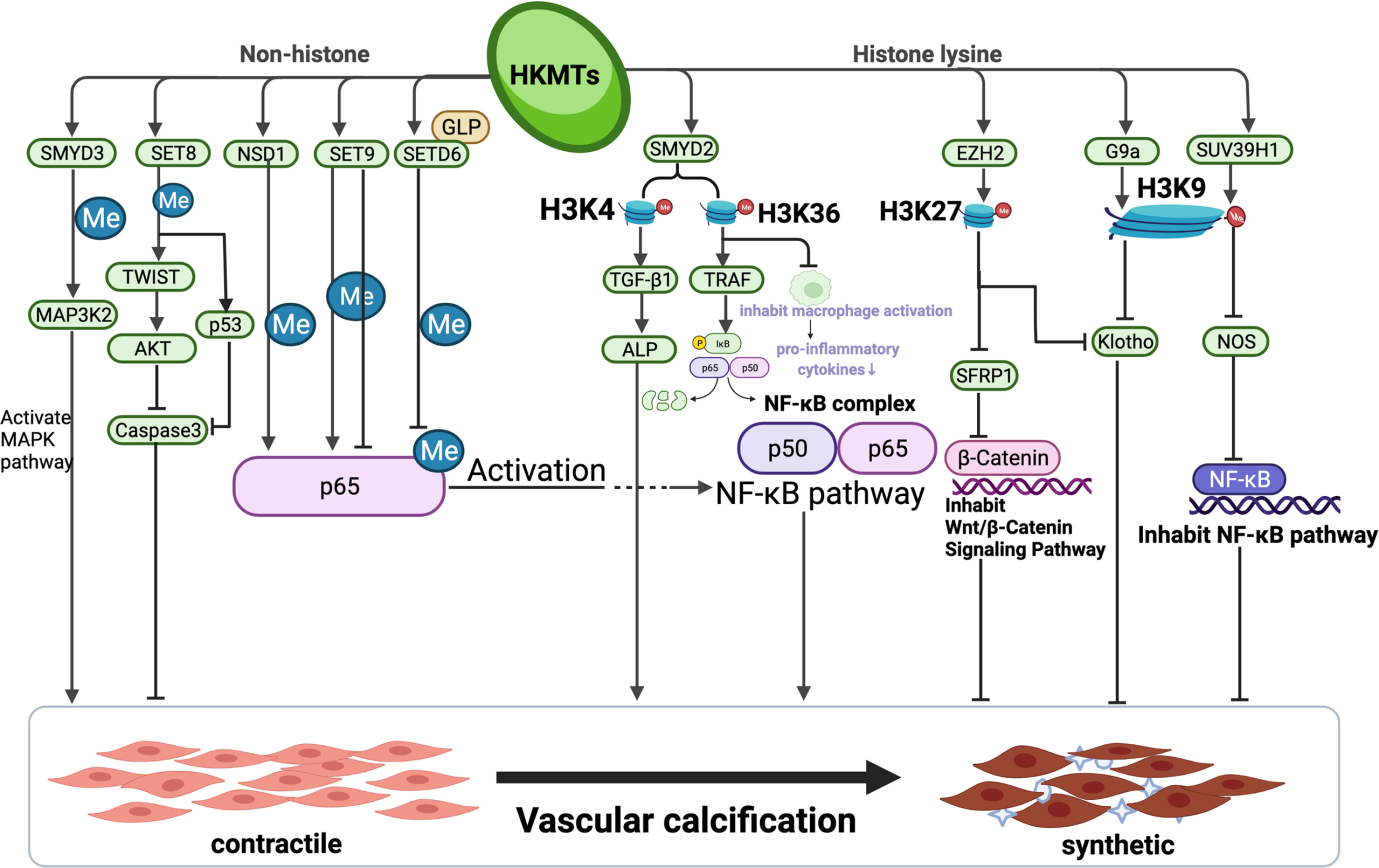
Complex network of relevant pathways involving mutual interactions in vascular calcification *via* HKMTs. The entire process of vascular calcification is the consequence of a complex integrated effect which is determined by multiple pathways. HKMTs, as a post-translational modifying enzyme that can act on both histone lysine and catalyze lysine from non-histone, have been shown to perform a sophisticated function in inhibiting or promoting multiple pathways of VC progression. This figure summarizes the pathways constituted by the mechanisms involved in vascular calcification by HKMTs and their role in the process of vascular calcification. Created with BioRender.com.

As a classic saying goes, all roads lead to Rome. During pathologic processes, many signal pathways work independently to achieve the same goal. HKMTs have been demonstrated to play an important role in each of these pathways, either by inhibiting or boosting gene expression, or by targeting critical components of a signalling system implicated in physiopathological activity. The majority of current research regarding the role of HKMTs in pathway signalling–mediated disease development is concentrated on tumours, and evidence has shown that HKMTs and pathological pathways associated with VC have sophisticated cross-talk. HKMTs are involved in various pathological alterations such as vascular inflammation, atherosclerosis and VC, and play seemingly minor epigenetic modifying roles throughout the pathway. Because HKMTs are post-translational modifying enzymes, they can affect gene expression at almost every segment of the pathway. When all pathways leading to VC are linked together, each location of the pathway may behave as a possible target for HKMTs to act, either activating or inhibiting the VC process. At the macroscopic level, independent and distinct HKMTs may burst into a mighty flame from little sparks (**Figure 2**).

### 6.1 Wnt/β-Catenin Pathway

The Wnt/β-catenin signalling pathway is a cell/receptor context–dependent route to activate the nuclear functions of β-catenin and can activate the expression of target genes, and this pathway is involved in virtually all physiological or pathological mechanisms in a variety of organisms. In the classical Wnt signalling pathway, when the Wnt ligand binds to the FZD-LRP5-LRP6 co-receptor, it promotes the phosphorylation of glycogen synthase kinase 3β (GSK3β), thereby β-catenin can be prevented from being degraded. In addition, β-catenin accumulates stably in the cytoplasm before it enters the nucleus to bind to T-cell factor/Lymphoid enhancer-binding factor (Tcf/Lef), triggering target gene transcription (151). Multiple studies have suggested that the Wnt signalling pathway is involved in VSMC calcification (152, 153), and Hao et al. identified elevated expressions of β-catenin and Wnt-5a in VC (154). Meanwhile, abnormalities in Wnt signalling are associated with aberrant epigenetic modification mechanisms (155). The histone H3K27 methyltransferase EZH2 is abundant in Wnt promoters, according to genome-wide profiling studies (156). H3K27me3 is an important modification involved in Wnt/b-catenin pathways, acting as a marker of gene repression (157). EZH2-regulated H3K27me3 plays a negative role on the β-catenin promoter during the odontogenic differentiation of hDPCs (158), and Lu et al. indicated that activation of EZH2 can inhibit the expression of Wnt and BMP targets and that PRC2 dysfunction can elevate Wnt signalling, shown by genomic occupancy and transcriptomic analyses. Together, reducing EZH2 activity and H3K27me3 levels may induce demyelinating diseases (159). Researchers have recently begun to investigate the existence and significance of histone lysine methylation modifications in the Wnt/β⁃catenin pathway in investigations into the mechanisms of VC. Advanced glycation end products (AGEs) can activate the Wnt/β⁃catenin signalling pathway by binding to the receptor for advanced glycation end products (RAGE) on the cell membrane (160), and it has been shown that AGEs significantly stimulate the expression of osteopontin (OPN), osteocalcin (OC) and Runx2 mRNA in rat aortic smooth muscle cells (161), during which β-catenin and OPG gene expressions are upregulated, leading to the differentiation of VSMCs into osteoblasts and ultimately to the development of VC (162). RAGE induces the conversion of VSMCs to osteoblasts through activation of signalling pathways such as ERK (163), NF-κB (164) and Wnt (160); thus, participating in VC. AGEs decrease EZH2 expression in podocytes and, consequently, reduces H3K27me3, causing an upregulated expression of pathological factors and contributing to podocyte injury in diabetic kidney disease (165). EZH2 is required in Wilm’s tumour 1 (WT1)-mediated β-catenin inactivation *via* repression of secreted frizzled-related protein 1 (SFRP-1), which is a Wnt antagonist, and EZH2-mediated silencing of SFRP-1 is due to increased H3K27me3 at its promoter area(s) (166). H3K27 methyltransferase EZH2 represses Wnt genes directly to facilitate adipogenesis, and deletion of EZH2 eliminates H3K27me3 on WNT promoters and de-represses Wnt expression, leading to activation of Wnt/beta-catenin signalling (167). The mechanism of SET8 activation of the Wnt signalling pathway is yet to be revealed, despite numerous studies (104). However, after activation of the Wnt signalling pathway, SET8 has been found to act as a transcriptional activation cofactor for H4K20me1 modification of histones, regulating chromosomal conformation and thus recruiting more transcription factors to accumulate and initiate downstream gene transcription (105). Through these findings, it is evident that the Wnt/β-catenin pathway plays an essential role in vascular calcification, as well as that HKMTs regulate VC progression by methylating critical factors in the pathway. Considering that HKMTs have a bidirectional effect on the regulation of the pathway, we couldn’t help wondering which modality of regulation is more beneficial in reducing the occurrence of vascular calcification?

### 6.2 NF-κB Pathway

In normally unstimulated conditions, nuclear factor kappa B (NF-κB) in the cytoplasm is inactivated and binds to inhibitor of kappa B proteins (IκBs), forming a trimeric complex. In the presence of external stimuli, TNF receptors on the cell membrane surface bind cytokines and multimerize, and then interact with TRADD molecules in the cytoplasm. IκB then dissociates from the p50/p65/IκB heterotrimer and is degraded; thus, NF-κB is released from repression and enters the nucleus, where it binds to specific sequences on intranuclear DNA to initiate or enhance the transcription of related genes (168). NF-κB signalling has been implicated in osteogenic differentiation of VSMCs in response to diverse stimuli such as high glucose (169), high phosphate (9, 170) and oxidative stress (171), as well as pro-inflammatory cytokines. TNF–NF-κB signalling can significantly increase ALP activity and TNF-activated NF-κB promotes inflammation-accelerated VC (172). In addition to enhancing ALP activity, NF-κB activation also upregulates BMP-2 and Runx2 expression, thereby interfering with the anti-calcification pathway in VSMCs (172). Barroso et al. revealed that EZH2 suppression promoted the expression of inflammatory cytokines by the reduction of H3K27me3 in promoters of related genes, and activated the NF-κB pathway in the vasculature (173). For example, the decrease of H3K27me3 at the IL-1β promoter can increase IL-1β expression, which acts on IL-1βR to activate the NF-κB pathway (174). EZH2 deficiency enhances tumour necrosis factor receptor-associated factor 2 (TRAF2) expression, thereby enhancing TNF-α-induced NF-κB signalling (175). SMYD2-mediated TRAF2 methylation continuously activates the NF-*κ*B signalling axis by sustaining its own stability (176). In addition to the amplified inflammatory program caused by inhibition of EZH2, inhibition of G9a expression can also enhance the NF-κB pathway by reducing H3K9me2 expression, inducing an inflammatory response in VSMCs (177). Therefore, increasing H3K9me2 or H3K27me3 levels of NF-κB pathway–associated sites, by regulating HKMTs’ activity or inhibiting relative demethylases, may provide a novel target strategy for VC.

### 6.3 BMP Pathway

BMP signalling exists mainly in the form of specific binding of ligands to serine/threonine kinase receptors on the cell membrane, forming a ligand-receptor binary complex. The type I receptors phosphorylate Smad proteins (Smad1, Smad5, and Smad8), prompting Smad molecules to detach from the cell membrane and enter the nucleus after binding Smad4 molecules (common-Smad, Co-Smad) in the cytoplasm. In the nucleus, the Smad multiplex acts on specific target genes with the participation of other DNA-binding proteins, regulating the transcription of the target genes (178). Researchers have confirmed since decades ago that BMP signalling was involved in VC (179) and that the BMP signalling pathway cross‐talks with the Wnt signalling pathway (180). SMYD2 was shown to have a strong association with the BMP pathway by methylating BMPR2, which, in turn, facilitates Smad1/5 phosphorylation, nuclear entry, and interaction with Smad4 and, consequently, BMP target gene expression (181). EZH2 deletion increases the BMP-dependent Smad1/5 phosphorylation by decreasing H3K27me3 near transcriptional start sites (182). Suv39h2 interacts with Smad5, and can silence the myogenic promoters by methylation of histone H3K9 which induces the expression of osteoblast-specific genes (183). Wang et al. described the reduced level of H3K9me3 and H3K27me3 and their occupancy at promoters of Bmp2 and Bmp4 without affecting the expression of HKMTs, implying that histone demethylases may be responsible for the reduction in methylation (184). In summary, according to the most recent findings, HKMTs act at several levels of the BMP signalling pathway, and BMP is regarded as a major contributor in VC promotion. Researchers should investigate other undiscovered mechanisms of the BMP pathway in VC advancement, as well as ways to control VC progression by controlling HKMTs’ activation or inhabitation.

### 6.4 PI3K/AKT Pathway

PI3K/AKT signalling plays a critical role in cellular physiology (185) and it is confirmed that calcification of VSMCs could be significantly reduced by applying LY294002, a specific inhibitor of the PI3K/AKT pathway, indicating that activation of the PI3K/AKT pathway could promote VC (186). One of the pathways of AGEs that lead to VC includes activation of the PI3K/AKT signalling pathway (187). Researchers have also identified that AKT can increase the stability and transcriptional activity of Runx2 protein to regulate osteoblast differentiation (188). In addition, PI3K/AKT and ERK signalling pathways can elevate BMP-4 and ALP expression through activation of RANKL, and this process can be reversed after pretreatment with LY294002 (42). The mechanism of interaction between the PI3K/AKT pathway and histone lysine methylation has also been widely studied. EZH2 can act as a substrate for AKT, and the activation of AKT signalling pathway phosphorylates EZH2, which decreases the affinity between EZH2 and histone, leading to the loss of EZH2 methylation histone function and the decrease of H3K27me3 level, promoting the development of disease (189). These discoveries demonstrate that the interaction of the PI3K/AKT pathway with altered methylation catalyzed by HKMTs has critical properties for VC progression. In conclusion, enhanced understanding of the role of HKMTs in the PI3K/AKT pathway affecting VC helps to enhance the physiological and pathological interpretation.

## 7 HKMTs Regulator and the Clinical Application Prospects in VC

Given that HKMTs-mediated histone or non-histone lysine methylation plays an important role in various pathways of VC development, we can investigate how targeting HKMTs or demethylases through small molecules can affect enzyme function, which may be an efficient therapy for VC. Since the discovery of histone lysine specific demethylase 1 (LSD1) (190), the thought that histone methylation is an irreversible process has been overturned, and researchers have begun to recognize that the histone methylation process is dynamically active, so how to use this dynamism to regulate disease onset and progression deserves the attention of researchers. Based on the previous studies, we expect mis-regulated genes can be re-expressed after treatment with methyltransferase inhibitors or activators in vascular calcification. A variety of researches have shown that inhibitors of HKMTs can be used in cancer treatment, for example, Tazemetostat, an EHZ2 inhibitor, for which researchers have started clinical trials to prove its efficacy in tumors such as lymphoma (191). However, few studies have investigated the treatment of VC by targeting methylated lysine associated with HKMTs, and the epigenetic regulatory medicines that researchers have identified that may be applied to control VC include inhibitors of histone deacetylase and histone acetyltransferases (192, 193). To date, these epigenetic mechanism-related small molecule inhibitors or activators have not been applied to clinical trials for the treatment of VC and related cardiovascular diseases. Therefore, researchers can focus on molecules which can regulate HKMTs and develop new epi-drugs targeting HKMTs to maintain the physiological homeostasis of the organism. It is expected to find an effective way to target VC through histone methylation modifications.

In addition, another application of epigenetic modifications in VC can be the determination of disease progression and prognosis by specific biomarkers. The current research indicates that methylated lysine sites such as H3K4me3 can be identified as epigenetic marks associated with Myh7 gene expression (194). And EZH2 has been proven to be a promising therapeutic and prognostic biomarker for tumors (195). In summary, investigators have confirmed in studies on other diseases that HKMTs and methylated substrates can be used as biomarkers for diagnosis and prognosis of diseases. Although there are no research results so far to suggest that HKMTs can be used as biomarkers for VC, it also gives researchers ideas to identify new markers that can contribute to the diagnosis and prognosis of VC.

## 8 Conclusion

In summary, histone lysine methylation modification is an important component of epigenetics, and their abnormal expression and function are receiving increasing attention with regard to the pathogenesis of VC. The study of histone lysine methylation in relation to VC has also made initial progress, showing that histone lysine methylation is associated with transcriptional activation or repression in different conditions in VC, and inhibiting or activating the expression of HKMTs can affect the progression. However, the exact mechanism of histone methylation modification is not well understood to date. Therefore, although abnormal histone lysine methylation has been verified to be associated with VC, further research is needed to elucidate the relationship between the two phenomena at the molecular level. An in-depth study of the pathogenesis of VC in combination with the characteristics of HKMTs will help to gain a deeper understanding of the “bridge” between histone lysine methylation modification and VC. The development of effective prevention and treatment tools based on this mechanism will be of great significance in controlling the development of VC and reducing mortality in the elderly, especially in patients with concomitant cardiovascular disease.

## Author Contributions

L-QY wrote the manuscript and approved the final version of the manuscript. Y-CC contributed to study conduct, data analysis, and manuscript writing. S-KS, BG, C-CL, F-X-ZL, M-HZ, Q-SX, YW, L-ML, K-XT, W-LO-Y, J-YD, Y-YW, MU, Z-AZ, FX, X-BL, FW and XL contributed to data analysis. All authors reviewed the manuscript. All authors contributed to the article and approved the submitted version.

## Funding

This work was supported by funding from the National Natural Science Foundation of China (Nos. 81770881 and 82070910). Key R & D plan of Hunan Province (2020SK2078). Natural Science Foundation of Hunan Province (2021JJ30036).

## Conflict of Interest

The authors declare that the research was conducted in the absence of any commercial or financial relationships that could be construed as a potential conflict of interest.

## Publisher’s Note

All claims expressed in this article are solely those of the authors and do not necessarily represent those of their affiliated organizations, or those of the publisher, the editors and the reviewers. Any product that may be evaluated in this article, or claim that may be made by its manufacturer, is not guaranteed or endorsed by the publisher.
